# Characteristics of somatic tinnitus patients with and without hyperacusis

**DOI:** 10.1371/journal.pone.0188255

**Published:** 2017-11-21

**Authors:** Massimo Ralli, Richard J. Salvi, Antonio Greco, Rosaria Turchetta, Armando De Virgilio, Giancarlo Altissimi, Giuseppe Attanasio, Giancarlo Cianfrone, Marco de Vincentiis

**Affiliations:** 1 Department of Oral and Maxillofacial Sciences, Sapienza University of Rome, Rome, Italy; 2 Center for Hearing and Deafness, University at Buffalo, Buffalo, New York, United States of America; 3 Department of Audiology and Speech-Language Pathology, Asia University, Taichung, Taiwan; 4 Department of Sense Organs, Sapienza University of Rome, Rome, Italy; University of Regensburg, GERMANY

## Abstract

**Objective:**

Determine if somatic tinnitus patients with hyperacusis have different characteristics from those without hyperacusis.

**Patients and methods:**

172 somatic tinnitus patients with (n = 82) and without (n = 90) hyperacusis referred to the Tinnitus Unit of Sapienza University of Rome between June 2012 and June 2016 were compared for demographic characteristics, tinnitus features, self-administered questionnaire scores, nature of somatic modulation and history.

**Results:**

Compared to those without hyperacusis, patients with somatic tinnitus and hyperacusis: (a) were older (43.38 vs 39.12 years, p = 0.05), (b) were more likely to have bilateral tinnitus (67.08% vs 55.56%, p = 0.04), (c) had a higher prevalence of somatic modulation of tinnitus (53.65% vs 36.66%, p = 0.02) and (d) scored significantly worse on tinnitus annoyance (39.34 vs 22.81, p<0.001) and subjective hearing level (8.04 vs 1.83, p<0.001).

**Conclusion:**

Our study shows significantly higher tinnitus modulation and worse self-rating of tinnitus and hearing ability in somatic tinnitus patients with hyperacusis versus somatic tinnitus patients without hyperacusis. These differences could prove useful in developing a better understanding of the pathophysiology and establishing a course of treatment for these two groups of patients.

## Introduction

Hyperacusis is a term used to describe intolerance to certain everyday sounds that causes significant distress and impairment in social, occupational, recreational, and other day-to-day activities [1]. The sounds may be perceived as uncomfortably loud, unpleasant, frightening, or painful [2,3]. Hyperacusis is often associated with the phantom sound of tinnitus [4–6].

While the neural mechanisms underlying hyperacusis are still unclear [3], there is growing awareness that hyperacusis may be related to increased neural gain at many different levels of the central auditory system and areas outside the classical auditory pathway involved in arousal, emotional response to sound, anxiety, stress and motor control [7–9]. Recent brain-imaging studies have identified neural hyperexcitability of certain areas of the brain both within and outside the classically defined auditory pathway [9–12]. Hyperacusis is sometimes associated with disordered perceptions involving the visual and somatosensory domains such as heightened sensitivity to light, migraine and lowered pain thresholds in individuals with chronic pain [13,14]. Hyperacusis is also associated with anxiety, depression, schizophrenia and sleep disorders [3,15,16]. Approximately 40% of patients with tinnitus also suffer from hyperacusis whereas up to 80% of patients with hyperacusis also have tinnitus, suggesting that these disorders may share a common pathophysiology [8,9,17]. While hyperacusis and tinnitus are often associated with hearing loss [18–20], some individuals with hyperacusis and/or tinnitus have clinically normal audiograms [15,21].

Somatic tinnitus, which affects a significant percentage of tinnitus sufferers [22,23], refers to a subtype of tinnitus that appears to be linked to an underlying somatic disorder and therefore related to problems of the musculoskeletal system rather than just the ear [22,24]. These results suggest some involvement or interaction of the musculoskeletal system with the central or peripheral auditory pathways [25–29]. Some studies have shown that patients with somatic tinnitus may have a higher prevalence of modulation of tinnitus loudness and pitch by movement of the head, neck, eyes and upper torso compared to the general tinnitus population, although this is still debated [28,30–38]. Electrophysiological and neuroanatomical studies have provided insights on the anatomical pathways through which the visual, somatosensory and motor systems can interact with the auditory system [22,24–35]; clinical studies have explored the association between somatic disorders and tinnitus [36–41]. This suggest that identifying disorders of the head, neck and upper torso could be clinically relevant to the management and treatment of tinnitus by non-auditory clinicians such as physical therapists.

Many studies have focused on the association between hyperacusis and tinnitus [8,9,17–20,42–44]; however, much less is known about the association between hyperacusis and somatic tinnitus, although the former has been shown to be associated with disordered perceptions involving the somatosensory domain. Some report an increased prevalence of hyperacusis in somatic tinnitus patients [45] while others have not [46]. It is unclear from the literature if somatic tinnitus patients with hyperacusis (ST+HY) have the same phenotypic characteristics as somatic tinnitus patients without hyperacusis (ST) or if they exhibit substantially different characteristics. To address this question, we compared ST+HY patients with ST patients on the following measures: demographics, tinnitus perceptual characteristics, self-administered questionnaire scores, somatic modulation features and history of somatic disorders.

## Materials and methods

This study included 172 clinically normal hearing patients with somatic tinnitus evaluated at the Tinnitus Unit of Sapienza State University in Rome, Italy from June 2012 to June 2016. Patients were divided into two groups: ST+HY patients (n = 82) and ST patients (n = 90).

Clinically normal hearing was defined according to the American Academy of Otolaryngology and American Council of Otolaryngology [47] as an individual hearing threshold ≤25 dB HL at frequencies from 250 to 4,000 Hz at the octave scale in both ears. Somatic tinnitus was defined by a positive history for temporomandibular joint (TMJ) and/or head and neck (NECK) dysfunction [48] and/or a positive modulation of tinnitus following somatic maneuvers [31]. Hyperacusis was defined by scores on the Khalfa’s Hyperacusis Questionnaire (HQ) [49] and Geräuschüberempfindlichkeit (Noise Hypersensitivity) (GUF) questionnaires (see below).

Exclusion criteria were hearing loss in at least one ear, middle or inner ear disease (e.g., otosclerosis, chronic suppurative otitis media or endolymphatic hydrops), retrocochlear disease (e.g., vestibular schwannoma), previous ear surgery, pulsatile tinnitus, concurrent medical treatment for tinnitus (e.g., sedatives, antidepressants) except for antioxidant drugs. All patients signed a written informed consent. The procedures performed were in accordance with the ethical standards of the responsible committee on human experimentation of the Department of Sense Organs, Sapienza University of Rome (ID714) that specifically approved this study and with the Helsinki Declaration [50].

Patients underwent an anamnestic interview, a full ear, nose and throat examination, an audiological test battery including pure tone audiometry (PTA) and acoustic immittance test, and somatic TMJ and NECK maneuvers. History of acoustic trauma or prolonged noise exposure was investigated during anamnestic interviews. PTA was measured at frequencies of 0.125, 0.25, 0.50, 0.75, 1, 2, 3, 4, 6, and 8 kHz; hearing was considered symmetrical if thresholds for each ear occurred within 10 dB of each other. Subjects completed the Italian versions of the Tinnitus Handicap Inventory (THI) [51], Hearing Handicap Inventory (HHI) [52], HQ [53] and GUF [54] questionnaires. The tinnitus characteristics assessed in the study were: tinnitus location (side, unilateral or bilateral) and tinnitus spectrum from a predefined set of possibilities including “buzzing”, “whistle”, “high-pitched”, “low-pitched” and “other”.

Hyperacusis was investigated with HQ and GUF questionnaires. A score equal or greater than 28 at HQ [53] and 16 at GUF [54] has been previously suggested to represent a strong auditory hypersensitivity. Patients were included in the hyperacusis group if their score equaled or exceeded 28 on the HQ and/or 16 on the GUF questionnaire.

Somatic tinnitus was determined from the history for previous somatic disorders and assessment of the patient’s ability to modulate their tinnitus. History for TMJ and/or NECK dysfunction was considered positive if one or more of the following events occurred within one year before the onset of tinnitus: head or neck trauma, intensive manipulation of teeth or jaw or cervical spine, recurrent pain episodes in head, neck or shoulders, increase of both pain and tinnitus at the same time, inadequate postures during rest, walking, working or sleeping, intense periods of bruxism during day or night [48]. Nineteen somatic head and neck maneuvers (Table 1) were performed to investigate if they elicited changes in tinnitus loudness modulation (increase/decrease). Patients were asked to perform a specific movement or to resist pressure applied by the examiner against the head, neck and jaw. Each contraction was held for 10 seconds. If the assessment resulted in tinnitus modulation, the examiner waited for tinnitus to return to baseline levels before proceeding with another maneuver. Tinnitus modulation was considered present if the patient reported tinnitus modulation following at least one of our somatic maneuvers.

**Table 1:** Somatic maneuvers.

**Jaw Maneuvers**		
TMJ 1	Clench teeth together	performed by patient
TMJ 2	Open the mouth with restorative pressure	performed by patient
TMJ 3	Protrude jaw with restorative pressure	performed by patient
TMJ 4	Slide jaw to left with restorative pressure	performed by patient
TMJ 5	Slide jaw to right with restorative pressure	performed by patient
**Neck maneuvers**		
NECK 1	Resist pressure applied to the forehead	performed by examiner
NECK 2	Resist pressure applied to the occiput	performed by examiner
NECK 3	Resist pressure applied to the vertex	performed by examiner
NECK 4	Resist pressure applied under the mandibule	performed by examiner
NECK 5	Resist pressure applied to the right temple	performed by examiner
NECK 6	Resist pressure applied to the left temple	performed by examiner
NECK 7	Pressure to the right zygoma with head turned right	performed by examiner
NECK 8	Pressure to the left zygoma with head turned left	performed by examiner
NECK 9	Pressure to the left temple with head turned right and tilted to the left (left sternocleidomastoid muscle)	performed by examiner
NECK 10	Pressure to the right temple with head turned left and tilted to the right (right sternocleidomastoid muscle)	performed by examiner
NECK 11	Forward flection of the neck	performed by patient
NECK 12	Backward flection of the neck	performed by patient
NECK 13	Turn head to the right	performed by patient
NECK 14	Turn head to the left	performed by patient

Maneuvers used for somatic testing in our study [31].

### Statistical analysis

To assess differences between the ST+HY and ST patients in terms of demographic characteristics, tinnitus characteristics, self-administered questionnaires and somatic modulation and history, a logistic regression analysis was performed. The logistic regression quantified the risks associated with the outcome of interest and potential risk factors such as demographics, tinnitus characteristics, and somatic modulation history. Both univariate and multivariate analyses were performed. In the univariate analysis, factors have been considered one at a time to fit the logistic regression model. In the multivariate analysis, all variables that were statistically significant in the univariate analysis were included. Results are reported as 95% confidence interval of odds ratio. The p-value for assessing statistical significance was an alpha of 0.05.

## Results

### Demographic, hearing and tinnitus characteristics

Results were obtained from 172 patients; 101 males (58.72%) and 71 females (41.27%). The demographic characteristics and questionnaire results are presented in Table 2. In the ST+HY group 54.87% were males and the mean age was 43.38 years (range: 17–69 years). In the ST group 62.22% were males and the mean age was 39.12 years (range: 18–66 years). Individuals in the ST+HY group were significantly older compared to the ST group (p = 0.05). Average PTA thresholds in the clinical audiometric range (0.25–8 kHz) were 16.7 dB HL (0.125–2 kHz), 24.5 dB HL (2–4 kHz) and 28.2 (4–8 kHz) with no significant interaural asymmetries.

**Table 2:** Tinnitus characteristics and questionnaire scores.

	ST+HY (n = 82)	ST (n = 90)	p-value
**Tinnitus Characteristics**			
Age	43.38	39.12	0.05
Gender			
Male	54.87%	62.22%	0.09
Female	45.13%	37.78%	
Lateralization			
Unilateral	32.92%	44.44%	0.04
Bilateral	67.08%	55.56%	
Tinnitus sound			
Whistle	37.80%	38.88%	0.64
Buzzing	20.73%	18.88%	0.76
High-pitched	17.07%	16.66%	0.82
Low-pitched	7.31%	20.00%	0.006
Other	17.07%	5.58%	0.01
**Questionnaires**			
THI score			
Severe (58–100)	21.95%	3.33%	<0.001
Moderate (38–56)	25.60%	7.77%	0.004
Mild (18–36)	42.68%	42.22%	0.72
No-handicap (0–16)	9.75%	46.67%	<0.001
Average			
HHI score	8.04	1.83	<0.001
Hyperacusis score			
HQ	26.36	5.45	<0.001
GUF	12.36	3.69	<0.001

Comparison of tinnitus characteristics and self-administered questionnaire results in our groups.

Average duration of tinnitus at the time of first admission was 3.22 years, with no significant differences between groups (p = 0.06). Tinnitus was bilateral in 61.05% of patients and unilateral in 38.95% of patients. In the ST+HY group 32.92% patients had unilateral tinnitus compared to 44.44% in the ST group; the difference was statistically significant (p = 0.04). “Low Pitched” tinnitus was less common in ST+HY group (7.31%) compared to the ST group (20%) (p = 0.006).

Logistic regression analysis indicated that: (a) ST+HY patients were 1.02 time more likely to be older than ST patients; (b) males were 0.59 time less common in the ST+HY group than the ST group; and (c) ST+HY patients were 2.51 times more likely to have bilateral tinnitus than ST patients (Table 3). In the univariate analysis, all variables showed statistical significant results whereas in the multivariate analysis no statistical significance was found.

**Table 3:** Tinnitus, demographic and somatic disorder history characteristics among ST+HY patients.

	Un-adjusted (or univariate)			Adjusted (or multivariate)		
*Characteristics*	*Odds ratio*	*Confidence interval*	*p-value*	*Odds ratio*	*Confidence interval*	*p-value*
Age	1.02	0.99–1.04	0.07	1.03	0.98–1.08	0.25
Sex (males)	0.59	0.32–1.08	0.09	0.94	0.29–3.03	0.91
Tinnitus side (bil)	**2.51**	1.35–4.68	0.004	2.51	0.76–8.22	0.13
Duration	1.08	1.0–1.17	0.04	0.98	0.85–1.14	0.81
THI	1.05	1.03–1.08	<0.001	1.02	0.98–1.07	0.26
HHI	1.20	1.11–1.29	<0.001	1.09	0.98–1.22	0.12
TMJ (total)	**1.69**	1.22–2.33	0.002	1.79	0.93–3.42	0.08
NECK (total)	1.50	1.18–1.91	0.001	0.98	0.65–1.49	0.93

Logistic regression of tinnitus, demographic and somatic disorder history characteristics among ST+HY patients for both univariate and multivariate analyses. Factors most strongly prevalent in ST+HY patients are set in bold. In the univariate analysis, all variables show statistical significant results whereas the multivariate analysis showed no statistical significance.

### Questionnaires

**Fig 1:**
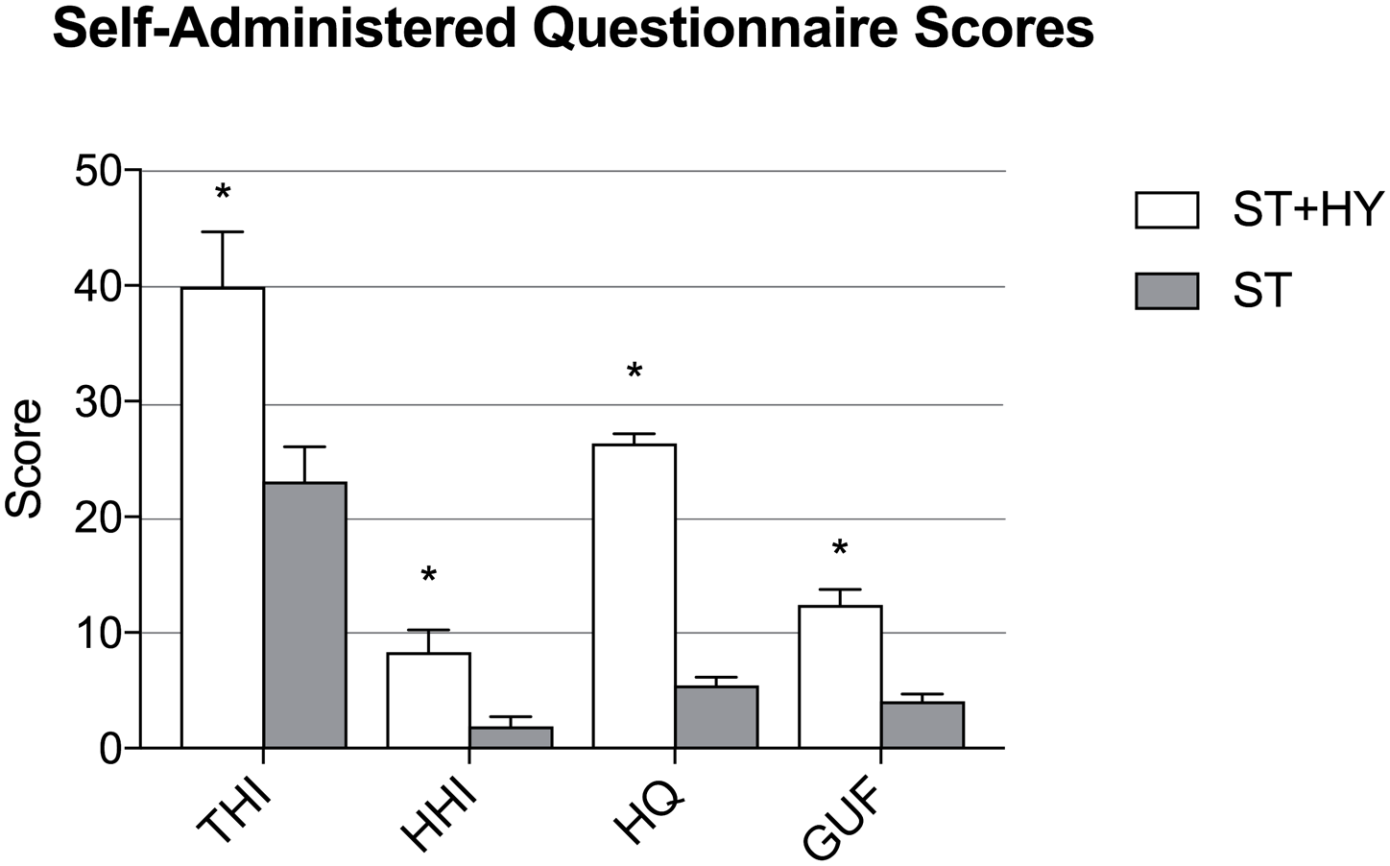
Self-administered questionnaire scores. Comparison of Self-Administered Questionnaire scores for Tinnitus Handicap Inventory (THI), Hearing Handicap Inventory (HHI), the Hyperacusis Questionnaire (HQ) and the Geräuschüberempfindlichkeit (GUF) questionnaires between hyperacusis (ST+HY) and non hyperacusis (ST) patients with somatic tinnitus. All questionnaire scores were significantly higher among ST+HY patients compared to ST patients (p<0.001).

Group differences in THI scores were as follows: no-handicap (THI = 0–16) in 9.75% of patients in the ST+HY group versus 46.67% in the ST group (p<0.001); mild tinnitus (THI = 18–36) in 42.68% of patients in the ST+HY group versus 42.22% in the ST group (p = 0.72); moderate tinnitus (THI = 38–56) in 25.60% of patients in the ST+HY group versus 7.77% in the ST group (p = 0.004); and severe tinnitus (THI = 58–100) in 21.95% of patients in the ST+HY group versus 3.33% in the ST group (p<0.001). Mean THI scores were significantly higher in the ST+HY group (39.34) compared to the ST group (22.81) (p<0.001). Group differences in mean HHI score were as follows: 8.04 in the ST+HY group versus 1.83 in the ST group (p<0.001). The mean HQ score was 26.36 in the ST+HY group versus 5.45 in the ST group (p<0.001). The mean GUF score was 12.36 in the ST+HY group compared to 3.69 in the ST group (p<0.001). In summary, all tinnitus, hearing handicap and hyperacusis questionnaire scores were significantly higher among ST+HY patients compared to T patients (p<0.001). However, it should be noted that the significant difference in hyperacusis questionnaire scores between the two groups was expected as this information was used as an inclusion criteria when allocating patients to one or the other group. (Fig 1).

### Somatic disorder history and modulation of tinnitus

In the ST+HY group 96.34% reported a positive history of somatic disorders compared to 88.88% in the ST with no significant differences between groups (p = 0.64). In the ST+HY group, 24.05% had a positive history for TMJ disorders, 17.72% for NECK disorders and 58.22% for both TMJ and NECK. In the ST group, 32.50% had a positive history for TMJ disorders, 26.25% had NECK disorders and 41.25% had both disorders.

In the ST+HY group, 53.65% of patients could somatically modulate their tinnitus whereas 36.66% of subjects in the ST group were able to do so; there was a significant difference between groups (p = 0.0095). In the ST+HY group, 29.54% could modulate their tinnitus following one or more TMJ maneuvers, 11.36% could modulate with one or more NECK maneuvers and 59.09% could modulate with one or more TMJ maneuvers and one or more with NECK maneuvers. In the ST group, 39.40% could modulate their tinnitus following one or more TMJ maneuvers, 33.33% with one or more NECK maneuvers and 27.27% with one or more TMJ and one or more NECK maneuver. Significantly more patients in the ST+HY group had a history (p = 0.05) and could modulate their tinnitus (p<0.001) for both TMJ-NECK compared to individuals in the ST group.

**Fig 2:**
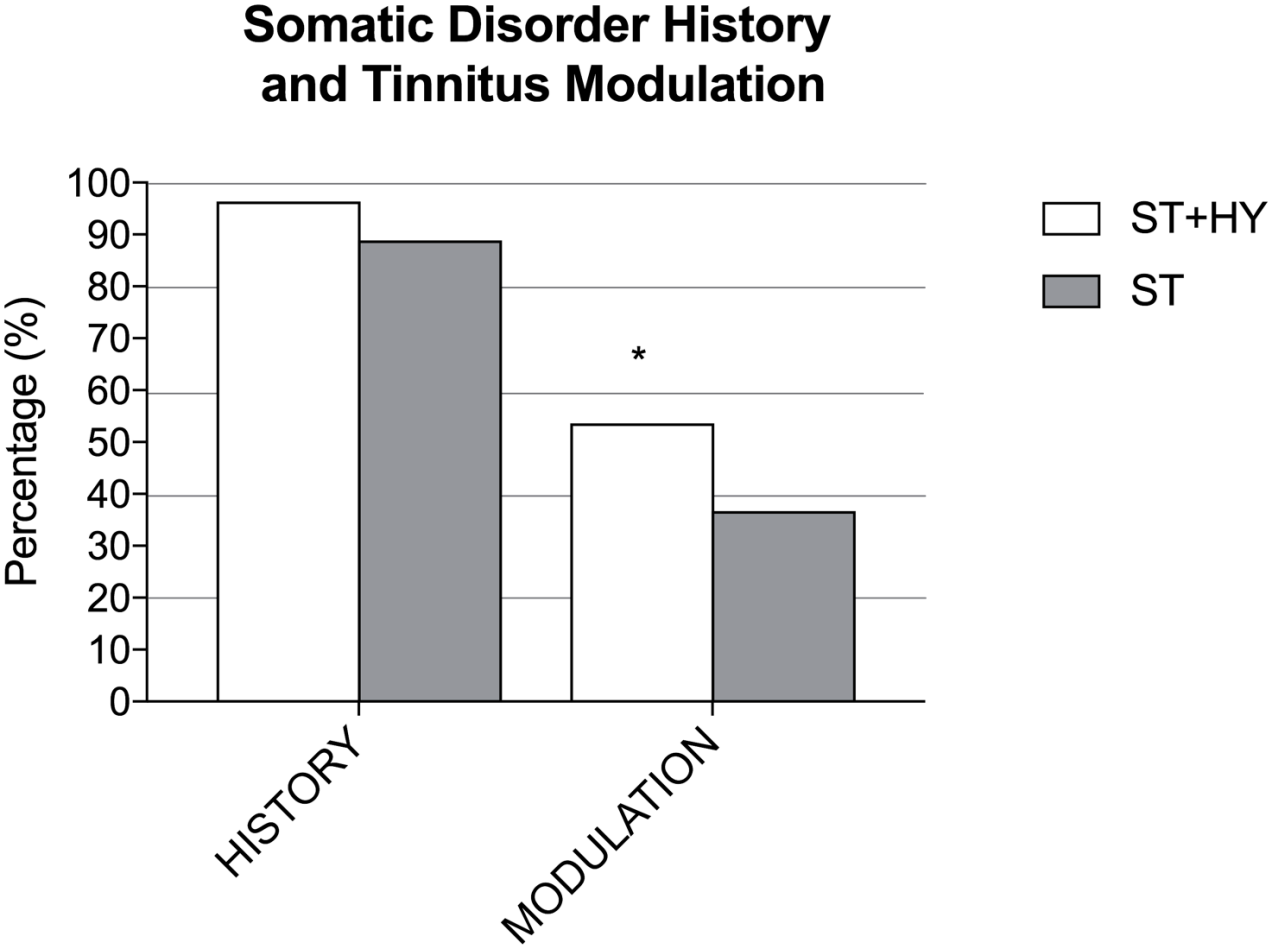
Somatic disorder history and tinnitus modulation. Percentages of somatic disorder history and somatic tinnitus modulation ability in somatic tinnitus patients with (ST+HY) and without (ST) hyperacusis. Compared to the ST group, significantly more patients in the ST+HY group could somatically modulate their tinnitus (p<0.001). No significant differences in history were found (p = 0.64).

**Fig 3:**
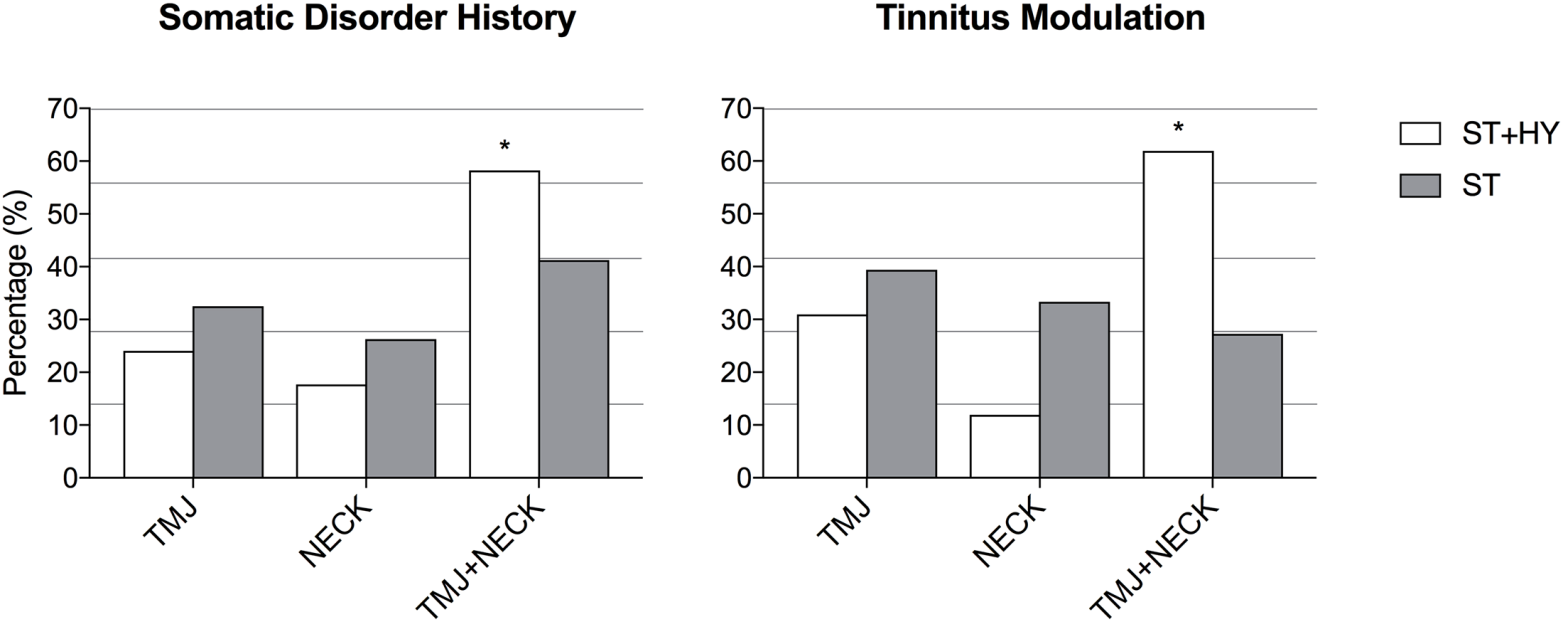
Comparison of somatic disorder history and tinnitus modulation. (A) Percentages of somatic tinnitus patients with (ST+HY) and without (ST) hyperacusis with temporomandibular joint (TMJ), head and neck (NECK) and TMJ+NECK problems among those with a history of somatic disorders. (B) Percentages of tinnitus modulation following TMJ, NECK and TMJ+NECK maneuvers among ST+HY vs. ST patients. TMJ+NECK history (p = 0.05) and modulation (p<0.001) were significantly more common in ST+HY than in ST patients.

Prevalence values for positive somatic history and positive tinnitus modulation in ST+HY and ST patients are shown in Figs 2 and 3.

A comparison between positive somatic history and positive somatic modulation of tinnitus with TMJ, NECK, and TMJ+NECK maneuvers among the ST+HY and ST patients is shown in Table 4.

**Table 4:** Comparison of somatic tinnitus history and modulation between groups.

	ST + HY GROUP (N = 82)		ST GROUP (N = 90)			
	History n (%)	Modulation n (%)	History n (%)	Modulation n (%)	*p-value (history)*	*p-value (modulation)*
TMJ	19 (24.05%)	13 (29.54%)	26 (32.50%)	13 (39.40%)	*0*.*43*	*0*.*88*
NECK	14 (17.72%)	5 (11.36%)	21 (26.25%)	11 (33.33%)	*0*.*24*	*0*.*12*
TMJ+NECK	46 (58.22%)	26 (59.09%)	33 (41.25%)	9 (27.27%)	*0*.*05*	*<0*.*001*
POSITIVE	79 (96.34%)	44 (53.65%)	80 (88.88%)	33 (36.66%)	*0*.*06*	*0*.*02*
NEGATIVE	3 (3.64%)	38 (46.35%)	10 (11.12%)	57 (63.34%)	*0*.*06*	*0*.*02*

Comparison between positive history and positive maneuver modulation in temporomandibular joint (TMJ), head and neck (NECK) and TMJ+NECK within somatic tinnitus + hyperacusis (ST+HY) and somatic tinnitus (ST) patients.

**Fig 4:**
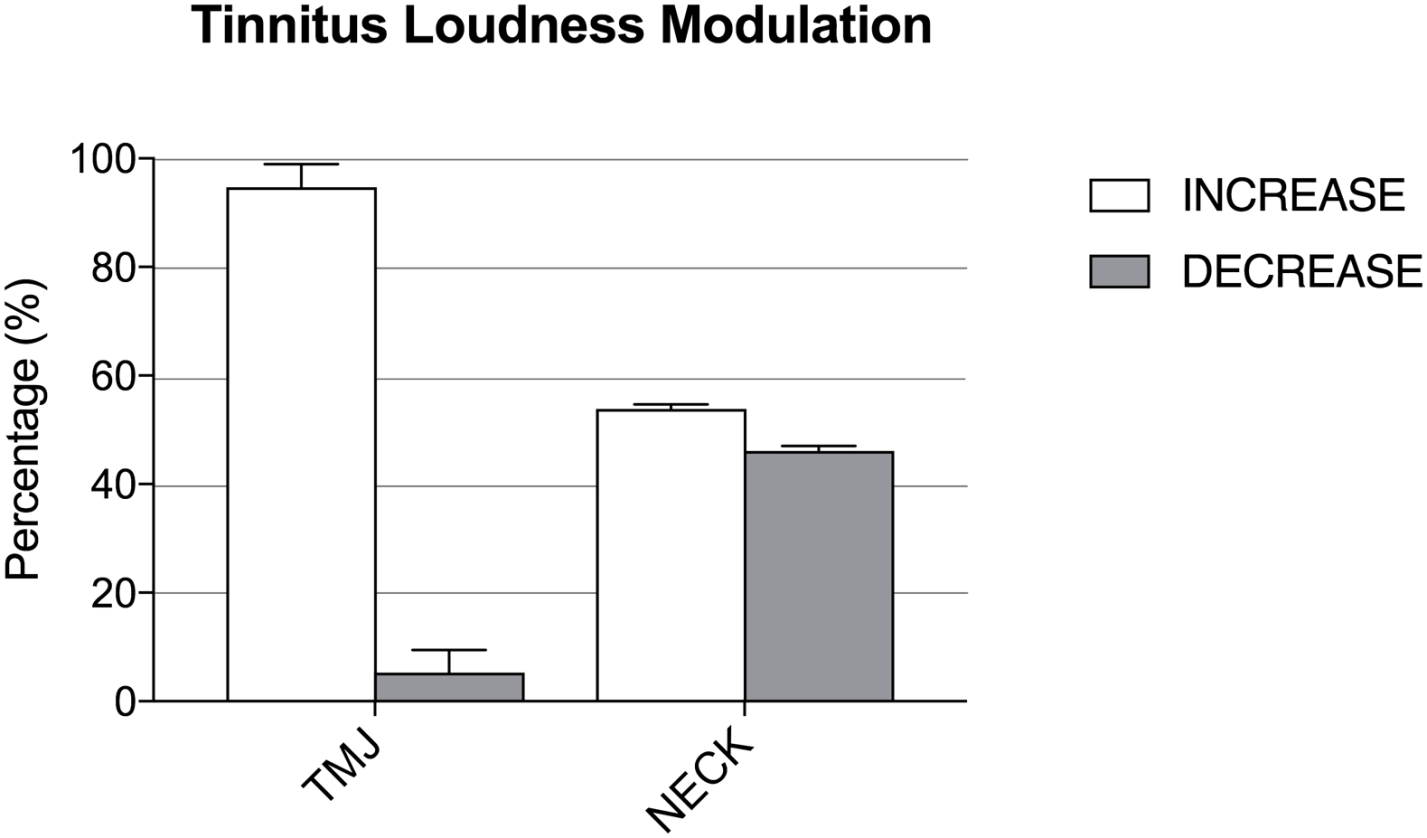
Tinnitus loudness modulation. Percentages patients that could increases or decrease the loudness of their tinnitus with temporomandibular joint (TMJ) or head and neck (NECK) maneuvers. Significantly more TMJ maneuvers increased tinnitus loudness (99.83% in the ST+HY group; 90.69% in the ST group) than decreased loudness (p<0.001). These findings are consistent with a previous study of our group on 310 tinnitus patients and with other authors who found a prevalent increase of loudness following TMJ maneuvers and a prevalent decrease following NECK maneuvers [31].

TMJ maneuvers generally resulted in increased tinnitus loudness in both groups (99.83% in the ST+HY group; 90.69% in the ST group), while a small portion caused a decrease in tinnitus loudness (p<0.001). NECK maneuvers resulted in an increase in tinnitus loudness in 54.45% of subjects in the ST+HY group versus 53.22% in the ST group, and a decrease in loudness in 45.55% of subjects in the ST+HY group versus 46.78% in the ST group (p = 0.87) (Fig 4).

## Discussion

The aim of this study was to compare patients with somatic tinnitus with and without hyperacusis on demographic variables, tinnitus characteristics, tinnitus questionnaire scores, somatic modulation of tinnitus and history of somatic disorders. Among patients with somatic tinnitus, those with hyperacusis were older, were more likely to have bilateral tinnitus, showed greater ability to modulate their tinnitus and scored significantly worse on self-administered questionnaires.

### Effects of hyperacusis on somatic tinnitus

We found a significantly higher percentage of somatic modulation of tinnitus in ST+HY patients versus ST patients. The largest difference was found for patients with involvement of both TMJ and NECK problems: 59.09% of individuals in the ST+HY group compared to 27.27% in the ST group. Our findings are in accord with Schecklmann [55]; they reported that somatic modulation occurred in 38% of ST+HY patients versus 27% of ST patients. The authors also reported that a history of TMJ disorder was present in 26% of their ST+HY group compared to 16% of ST patients; neck pain was present in 62% of ST+HY patients versus 48% of ST patients and more ST+HY patients had headaches and other musculoskeletal pain than ST patients. The study from Schecklmann, however, was not limited to patients with somatic tinnitus.

The increased prevalence of somatic modulation found in ST+HY patients versus ST patients could be due increased peripheral somatic activation or central hypersensitivity to somatic inputs in hyperacusis patients. The latter is supported by neurophysiological findings studies that report increased sensitivity to multisensory stimuli in hyperacusis patients, which may be linked to a hypervigilance network [12,16,56].

### Psychological correlates of hyperacusis

ST+HY patients rated their tinnitus as louder and more annoying and their hearing as worse compared to ST patients; the self-ratings suggest that psychological factors affect the self-perception of the disorders. Our findings are consistent with Schecklmann [55] and Gilles [57] who found worse self-perceived hearing ability, tinnitus and depression scores in patients with hyperacusis than those without. Higher tinnitus loudness, discomfort and annoyance could be explained by the involvement of emotion-related neural circuits. Juris [58] and Villaume [59] analyzed personality traits in hyperacusis patients and found a clear association between health-relevant personality traits and hyperacusis; there was a strong association between hyperacusis and negative affect. Specific personality traits, such as neuroticism are associated with depression [60], anxiety, panic [61] and negative impact on quality of life [62] and thus worse subjective health perception [63,64]. These results support the role of non-auditory areas in hyperacusis, such as the anterior cingulate and orbitofrontal cortex, known to be involved in vigilance and salience detection and pathologically involved in anxiety, hypervigilance and hyper-responsive behavior [9,55]. The higher scores in our ST+HY patients are also in agreement with the higher prevalence of psychiatric comorbidity in patients with high THI scores consistent with previous work from our group [65].

### Other phenotypic characteristics of hyperacusis patients with somatic tinnitus

Significant differences for age, tinnitus laterality and tinnitus pitch were found between the ST+HY and ST groups; the former were older and were more likely to have bilateral tinnitus. Our findings differ from others [55] who found that tinnitus characteristics were not related to hyperacusis. However, this difference may be related to the fact that our subjects had somatic tinnitus. However, it should be noted that while in the univariate analysis all variables showed statistical significant results, in the multivariate analysis no statistically significant results were found. These results suggest that there could be an impact of the characteristics on the case-control status irrespective of other variables in the univariate analysis; however, in the multivariate analysis this association was masked. The reason behind losing statistical significance in multivariate setting could be due to the correlation among the risk factors. If the potential risk factors are correlated among themselves, it should be expected that they lose statistical significance in a multivariate model, while the univariate analysis will explain the relation with the outcome of interest. Further studies on larger samples are necessary to understand if specific tinnitus characteristics are more common in ST+HY patients versus those with just tinnitus.

### Clinical implications

The association of tinnitus with somatic disorders has been reported previously [24,31–39,46,66–69], and improvements in tinnitus often occur after treatment of TMJ disorders [41,46], especially among those with a positive history for somatic disorders and modulation of the same somatic region [31]. In these patients, treatment of the somatic disorders could play a central role in alleviating tinnitus [23]. However, when patients present with both tinnitus and hyperacusis, additional factors may be involved. Our ST+HY patients show an enhanced reactivity for somatic modulation and self-administered questionnaires; these differences could prove useful in developing a better understanding of the pathophysiology and establishing a course of treatment for these two groups of patients, and should be considered when using somatic approaches to treat tinnitus in ST+HY patients.

### Considerations and limits of the study

Although hyperacusis is generally described as a reduced tolerance to sounds, hyperacusis inclusion criteria differ among studies. We relied exclusively on self-administered questionnaires to identify hyperacusis groups based on the criteria for the HQ and GUF questionnaires [53,54]. The threshold criteria, especially for HQ have been suggested as too strict [70,71]. In fact, there are controversies with regard to the cut-off score on HQ to be considered a reliable indicator for hyperacusis. Khalfa et al. [53] suggested a cutoff score of 28, Meeus et al. [70] suggested a cutoff of 26, while a more recent study from Aazh and Moore [72] suggested that a cut-off score of 22 on HQ offer a better match to reduced Uncomfortable Loudness Levels. Thus, the specific questionnaire and criteria used in our study may have biased our results to those with more severe hyperacusis.

Hidden and high-frequency hearing loss and its possible deafferentation origin for tinnitus [73] has not been studied in enrolled patients. Audiological analysis followed clinical guidelines and was performed up to 8 kHz; also, following our inclusion criteria, hearing ≤25 dB HL was considered normal. Given the spread of hidden hearing loss among general population, and especially among tinnitus sufferers and in subjects above the age of 40 [74–77], the presence of unexplored hidden hearing loss, especially in the 10–16 kHz range, should be considered in our patients.

The Italian versions of the hyperacusis questionnaires have been used in the present study. The HQ questionnaire has been validated in Italian by Fioretti et al. in 2011 [53]; however, the GUF questionnaire—although translated in Italian—has not been validated in the Italian language and is a potential limitation of our study.

There is still a controversy regarding the most appropriate criteria to diagnose somatic tinnitus. Some authors consider somatic modulation of tinnitus as an indicator for somatic tinnitus [66], while others consider it as a fundamental characteristic of tinnitus [22]. History for TMJ and/or NECK dysfunction, especially when the somatic event occurred before the onset of tinnitus, may be considered a valid indicator of the somatic origin of tinnitus [48]. A recent paper from Ralli et al. [31] reported a strong association between a positive history and modulation for the same somatic regions. This correlation suggested somatic disorder play an important role in tinnitus. The criteria adopted in the present paper to select somatic tinnitus patients relied on a positive history for somatic disorder and/or positive tinnitus modulation. The former was based on the definition of Sanchez et al. [48]; the latter on the recent work from Ralli [31].

## Conclusion

Our study shows significantly higher tinnitus modulation and worse self-rating of tinnitus and hearing ability in ST+HY patients versus ST patients. When evaluating somatic tinnitus patients, clinicians should consider that comorbid hyperacusis could amplify subjective somatic modulation of tinnitus, as well as self-perceived hearing ability, tinnitus loudness and annoyance and depression scores. Although the contribution of peripheral or central factors to hyperacusis is still unclear, there is growing recognition that hyperacusis may result from a generalized hypersensitivity disorder involving several sensory pathways and/or hypervigilance networks. Therefore, it is recommended to determine if hyperacusis is present in patients with somatic tinnitus, to judiciously select patients whose tinnitus would benefit from a somatic therapy.
